# Mechanistic Insights into SAM-Dependent Methyltransferases: A Review of Computational Approaches

**DOI:** 10.3390/ijms26189204

**Published:** 2025-09-20

**Authors:** Mateusz Jędrzejewski, Łukasz Szeleszczuk, Dariusz Maciej Pisklak

**Affiliations:** 1 Department of Organic and Physical Chemistry, Faculty of Pharmacy, Medical University of Warsaw, Banacha 1 Str., 02-093 Warsaw, Poland; mateusz.jedrzejewski@wum.edu.pl (M.J.); lukasz.szeleszczuk@wum.edu.pl (Ł.S.); 2 Doctoral School, Medical University of Warsaw, Żwirki i Wigury 61 Street, 02-091 Warsaw, Poland

**Keywords:** methyltransferases, QM-cluster, MD, QM/MM, reaction mechanisms

## Abstract

Methylation reactions catalyzed by S-adenosylmethionine (SAM)-dependent methyltransferases are essential to numerous biological functions, including gene expression regulation, epigenetic modifications, and biosynthesis of natural products. Dysregulation of these enzymes is associated with diseases, including cancer and neurodevelopmental disorders, making them attractive drug targets. This review explores the contribution of computational methods, particularly quantum chemical calculations and molecular dynamics (MD) simulations, in elucidating the mechanisms of SAM-dependent methyltransferases. These techniques enable detailed characterization of transition states and reaction pathways, often inaccessible by experimental methods. The review discusses molecular modeling approaches such as the quantum chemical cluster approach (QM-cluster) and hybrid QM/MM methods, emphasizing their applications in studying methyl group transfer, substrate specificity, and the roles of water molecules and metal ions in catalysis. Additionally, dynamic aspects of enzyme function are addressed using classical MD and QM/MM MD simulations. Case studies demonstrate how computational predictions align with experimental data and enable rational design of selective inhibitors and engineered enzymes with altered specificity. Overall, computational chemistry offers a powerful, atomistic view of SAM-dependent methyltransferases, not only complementing experimental studies but also providing a foundation for the design of future experiments in this field.

## 1. Introduction

**Figure 1:**
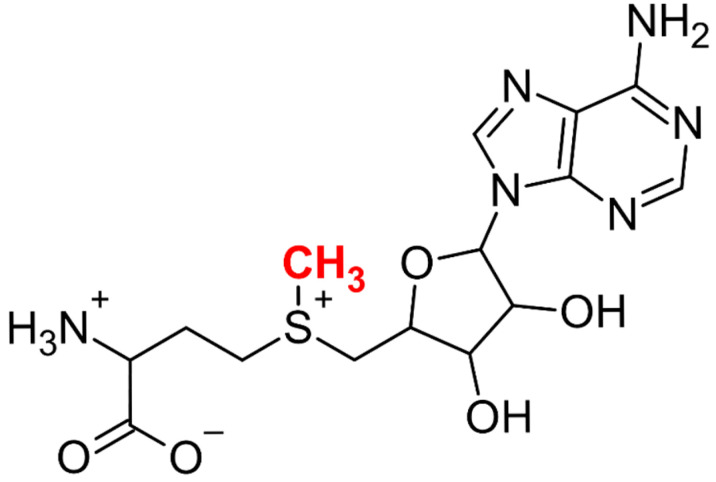
Structure of S-adenosylmethionine. The molecule consists of a methionine residue covalently linked to an adenosyl moiety, with the reactive methyl group highlighted in red.

Methylation is a fundamental biochemical reaction present across all domains of life. It is catalyzed by structurally diverse methyltransferases (EC 2.1.1), which participate in numerous biological processes [1] such as the biosynthesis of plant secondary metabolites [2], gene expression [3], signaling [4], genome repair [5], and drug biotransformation [6]. A majority of these enzymes rely on SAM as a methyl donor. SAM is an organic cation and the second most common enzymatic cofactor after ATP. Historically, the first enzyme with characterized methyltransferase activity was nicotinamide N-methyltransferase (NNMT), which was partially purified and described by Giulio Cantoni [7]. Although the nature of the methyl group donor was unknown at the time, Cantoni correctly hypothesized that NNMT uses a reactive compound formed from ATP and methionine as a cofactor. A year later, he identified SAM as a universal methyl group donor (Figure 1) [8].

SAM-dependent methyltransferases form a structurally and functionally diverse group. They are classified into five fold-based classes, with Class I (Rossmann fold) being the most common [9]. These enzymes act on a wide range of substrates, from small molecules to macromolecules such as tRNA, rRNA, DNA, and proteins, making them crucial for cellular homeostasis. Dysregulation of methyltransferase activity is linked to several diseases, including ICF syndrome, Fragile X syndrome, and various cancers, where abnormal DNA methylation patterns alter gene expression and genome stability. Given their central role in epigenetics, methyltransferases have become promising therapeutic targets. DNA methyltransferases, for example, are inhibited by hypomethylating agents such as azacitidine and decitabine in the treatment of acute myeloid leukemia. However, many SAM- or substrate-mimicking inhibitors lack specificity. To address this, bisubstrate analogs that mimic the transition state of the methylation reaction have been developed, offering much greater selectivity. This approach is exemplified by NTMT1 inhibitors, where some compounds showed over 3000-fold higher specificity for NTMT1 compared to other methyltransferases, including its close homolog NTMT2 [10].

In this review, we focus on how computational methods, particularly quantum chemical calculations and molecular dynamics simulations, contribute to understanding the mechanisms of SAM-dependent methyltransferases. Detailed knowledge of these enzymatic mechanisms, especially the structure of the transition state, is not only fundamental to elucidating enzyme function but also plays a key role in the rational design of selective and potent inhibitors. Since such short-lived states are usually inaccessible to experimental techniques, their characterization relies on computational approaches capable of capturing atomic-level details. This review summarizes current strategies used to model methylation reactions and highlights how mechanistic insights advance our understanding of methyltransferase function and reactivity. A comprehensive table summarizing the computational studies discussed in this review is provided in the Supplementary Materials, offering a structured overview of the relevant works.

### 1.1. Catalytic Mechanisms of SAM-Dependent Methyltransferases

**Figure 2:**
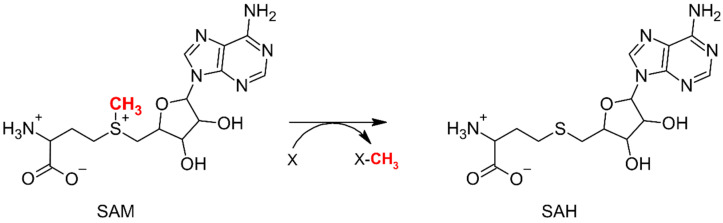
General mechanism of a reaction catalyzed by a SAM-dependent methyltransferase. In this process, the methyl group of S-adenosylmethionine (SAM) is transferred to the substrate, and the cofactor is converted into S-adenosylhomocysteine (SAH).

Methylation reactions catalyzed by SAM-dependent methyltransferases occur via an S_N_2 nucleophilic substitution mechanism [11]. In this process, the active methyl group attached to the sulfur atom of SAM is transferred to a nucleophilic site on the substrate, typically an oxygen, nitrogen, carbon, or sulfur atom (Figure 2). At the transition state, the methyl group adopts a planar, carbocation-like geometry and aligns linearly (or nearly so) with both the SAM sulfur and the substrate nucleophile, as required for the methyl group transfer reaction [12]. Numerous studies underscore the importance of hydrogen bonds between backbone carbonyl oxygens of active-site residues and the methyl group of SAM, which help stabilize this high-energy transition state [13,14,15]. In the case of methyltransferase-catalyzed reactions, three general reaction mechanisms can be distinguished [16]. The first mechanism, known as the proximity and desolvation mechanism, was proposed for methyltransferases in the SABATH family (salicylic acid, benzoic acid, and theobromine synthase). In this model, the enzyme’s active site is responsible for the proper orientation and proximity of the substrates needed for nucleophilic substitution. Additionally, the enzyme desolvates the reagent molecules, increasing their electro- and nucleophilicity [4]. The second mechanism involves acid/base catalysis, where specific amino acid residues in the active site facilitate the reaction by acting as general bases. This mechanism has been proposed, among others, for the NEP1 methyltransferase, where an aspartic acid residue deprotonates the substrate [17,18]. The third mechanism, involving metal cation-dependent methyltransferases, is prevalent mainly among phenolic O-methyltransferases in plants [19,20,21]. In this class of enzymes, metal ions, most commonly Mg^2+^ and Ca^2+^, play a direct role in catalysis.

These distinct catalytic strategies highlight the versatility of SAM-dependent methyltransferases in accommodating a wide variety of substrates and chemical environments. Understanding these mechanisms at a molecular level is crucial not only for elucidating enzyme function but also for guiding the rational design of selective inhibitors, which have therapeutic potential in diseases linked to aberrant methylation. Furthermore, insights into these mechanisms enable bioengineering efforts to develop methyltransferases with tailored specificities for applications in synthetic biology and drug development.

### 1.2. Why Use Computational Approaches?

**Figure 3:**
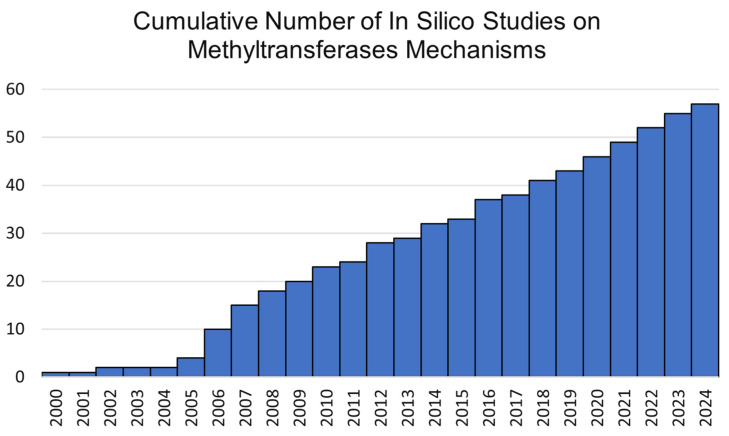
Cumulative number of in silico studies on methyltransferases over time, illustrating the general increasing trend in computational research on enzyme reaction mechanisms. The graph is based on selected representative publications listed in the table provided in the Supplementary Materials.

Experimental methods are widely recognised in the study of mechanisms of reactions catalysed by enzymes. Site-directed mutagenesis, enzyme kinetics, and isotope experiments, as well as crystal structure analysis, provide valuable details about the reaction mechanism [22,23]. Despite their undoubted advantages, these methods also have their limitations. For this reason, it seems helpful to use additional tools that allow information to be obtained that is difficult to access using experimental techniques. Due to the huge development of computing power and the increased availability of high-performance computing systems, there is a growing interest in the use of computer modeling methods in the analysis of biomolecular systems (Figure 3) [24]. They enable the study of enzymatic reaction mechanisms at the atomic level. Therefore, it is possible to characterize the course of a chemical reaction in detail, including the characterization of intermediates and transition states. The short lifetimes of these chemical individuals significantly hinder experimental analysis. Molecular modeling methods allow not only for the characterization of the structures of short-lived molecules but also for the determination of reaction thermodynamic parameters, such as the reaction energy barrier and the reaction free energy. Additionally, these techniques provide insight into atomic-level dynamics, enabling the observation of how atomic positions evolve throughout the reaction process.

Another advantage of computational methods in studying reaction mechanisms is that the state of the system is precisely known and can be controlled. This includes characteristics such as the protonation state, the presence of mutations or post-translational modifications, bound ligands, as well as the initial conformation of the enzyme. Such a high degree of control over the system enables the analysis of enzymatic reactions under varying conditions and facilitates the investigation of how these factors influence the reaction process and outcomes. Moreover, the application of computational methods aligns with the principles of green chemistry. Since these approaches do not require reagents, solvents, or laboratory infrastructure, they contribute to reducing the environmental impact of scientific research and the overall consumption of resources. This makes them a sustainable and eco-friendly complement to traditional experimental techniques.

In summary, molecular modeling methods offer detailed insight into enzyme-catalyzed reaction mechanisms at the molecular level, which is often difficult to obtain through experimental approaches alone. These computational techniques not only complement experimental data but also serve as a valuable tool for guiding the design of future experiments aimed at validating theoretical predictions.

### 1.3. Computational Approaches for Enzyme Mechanism Modeling

Molecular modeling methods can be classified in various ways, but they are typically categorized according to the level of detail in the representation of molecular systems. Depending on the approach, atoms or functional groups may serve as the smallest modeled units, as in molecular mechanics (MM) methods, or the system may be described at the level of nuclei and electrons, as in quantum mechanical (QM) methods. The main practical difference between these two types of methods relates to the speed and accuracy of the calculations.

MM methods are based on a significantly simplified description in which atoms are described as “balls” and bonds as “springs”. The potential energy of a system at the MM level is described by something we call a force field. A force field is a function and a set of parameters that describe interactions such as bond stretching, angle bending, dihedral rotations, as well as electrostatic and van der Waals forces. The most popular force fields for protein systems are AMBER [25,26,27,28,29], CHARMM [30,31,32,33,34,35,36,37,38,39,40,41,42,43,44,45,46,47,48,49], and OPLS [50,51], while for water molecules they are TIP3P [52], SPC [53], and SPC/E [54]. MM methods are characterized by very high computational efficiency, which makes it possible to analyze very large systems (hundreds of thousands of atoms). A significant limitation is their inability to describe the process of bond breaking and formation, as well as their significantly lower accuracy compared to QM methods. In recent years, machine learning force fields (ML-FFs) have been developed to bridge the gap between the accuracy of ab initio methods and the efficiency of classical force fields [55]. Instead of relying on predefined bonding patterns or interaction forms, ML-FFs model the statistical relationship between molecular structure and potential energy directly from reference quantum data. Their accuracy and efficiency depend primarily on the quality and amount of training data. Importantly, such ML-FFs can be integrated into multiscale schemes, offering a promising route to accelerate QM/MM molecular dynamics simulations by reducing the computational cost [56].

QM methods are based on approximate solutions to the Schrödinger equation, which allows for a separate description of atomic nuclei and electrons. One of the most used methods for analyzing enzymatic reaction mechanisms is density functional theory (DFT). It combines relatively high accuracy with moderate computational requirements compared to other QM methods. The most used DFT functional is B3LYP [57,58,59,60,61], and the most common basis sets are the Pople ones [62]. Basis sets define the group of mathematical functions used to describe atomic orbitals and are crucial for the quality of the result. For larger systems where standard DFT becomes computationally too demanding, the density functional tight binding (DFTB) method is often used as a compromise [63,64]. It is a simplified version of DFT that significantly reduces computational cost while maintaining a reasonable level of accuracy, making it useful for studying large biomolecular systems. Unlike MM methods, QM methods allow for the description of chemical bond formation and breaking, making them particularly suitable for analyzing reactions, including determining reaction barriers and describing transition states. The accuracy of the description offered by QM methods can be considered both a drawback and an advantage. QM methods are characterized by very high accuracy, but their significant computational complexity limits their application to small systems (from a few dozen to a few hundred atoms).

Considering the advantages and limitations of MM and QM methods, hybrid QM/MM approaches are also currently being developed. These methods combine the accuracy of QM calculations for the reactive region with the efficiency of MM for the surrounding environment, allowing for the study of chemical reactions in large biomolecular systems such as enzymes. This multiscale approach enables a more realistic representation of biological processes while maintaining reasonable computational cost.

#### 1.3.1. QM-Cluster and QM/MM Approaches

One of the first approaches in studying the mechanisms of enzymatic reactions was the quantum chemical cluster approach. In the QM-cluster approach, a carefully selected part of the enzyme, including the active site, is investigated using QM methods. DFT methods, particularly B3LYP functional and Pople basis sets, remain the standard computational choice. Models are usually based on crystallographic data, although the use of conformations derived from MD simulations is becoming increasingly common [17,65,66]. Initially, the models included no more than a few dozen atoms [67,68], but with the increase in the power of computers, it is now possible to analyze systems consisting of several hundred atoms [69,70,71]. However, considering that enzymes can consist of over a hundred thousand atoms, choosing this truncated system seems to be the most significant challenge in using the QM-cluster approach. In most studies, models are constructed based on chemical intuition and experimental data, such as mutation analysis, while research using more reproducible methods like the Residue Interaction Network-based ResidUe Selector (RINRUS) remains in the minority [70,72,73].

**Figure 4:**
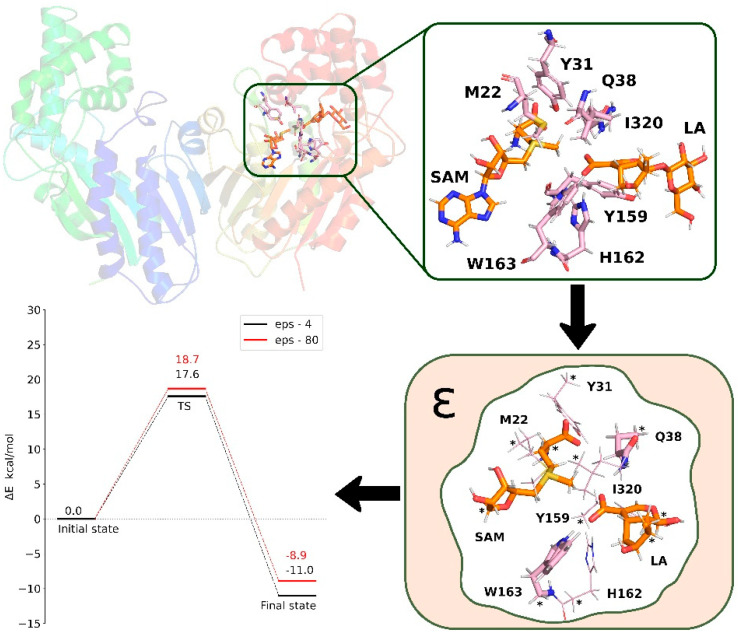
General steps of the QM-cluster approach for studying enzymatic reaction mechanisms. A selected fragment of the enzyme is extracted from the full complex structure. Peripheral atoms are fixed, and the influence of the surrounding protein is approximated using continuum solvent models. Quantum mechanical calculations are then performed on this truncated system to analyze the reaction pathway and energy barriers.

Electrostatic and steric effects of the omitted part of the enzyme are modeled approximately in the QM-cluster approach. To avoid unnatural movements of the amino acid residues, the atoms located at the periphery of the model are fixed in their initial positions. The electrostatic influence of the surrounding protein environment is typically accounted for by using a continuum solvent model with an assigned dielectric constant (typically, ε = 4). The most commonly applied models include polarizable continuum model (PCM) [74,75,76], conductor-like PCM (CPCM) [77,78], and conductor-like screening model (COSMO) [79]. The choice of a specific ε value might seem arbitrary, but in the case of large models, this choice becomes less significant since the majority of electrostatic interactions have been explicitly included in the model (Figure 4) [80,81,82].

The QM-cluster approach enables the analysis of the chemical step of an enzymatic reaction, starting from the structure of the enzyme–substrate complex. This methodology allows for the determination of the reaction energy barrier, reaction energy, and comparison of different reaction mechanisms. However, it is not possible to determine the free energy of binding of substrates/products, nor the influence of amino acid residues not included in the model. To analyze such aspects of enzymatic catalysis, it is necessary to use other computational methods.

**Figure 5:**
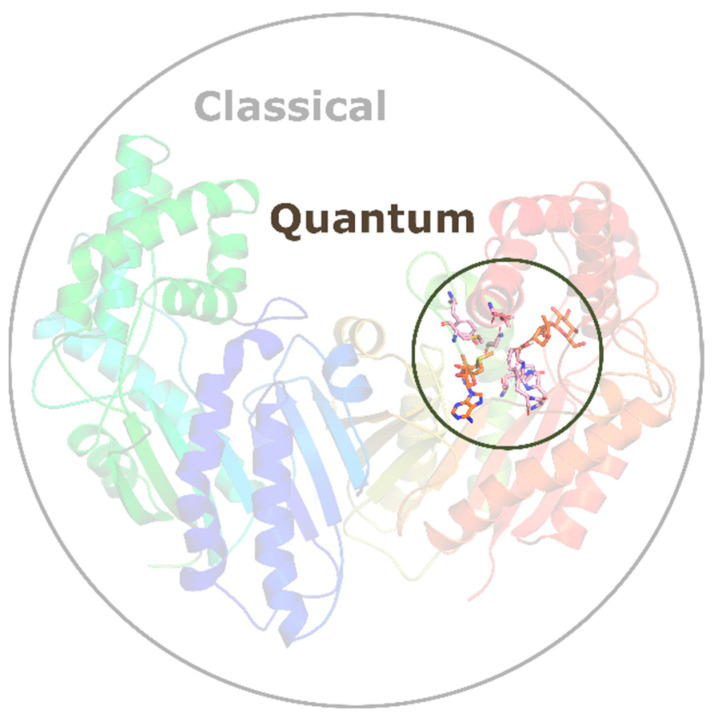
General idea of the QM/MM approach. Quantum mechanics is applied to the chemically active region, while molecular mechanics describes the surrounding environment.

An alternative approach in the analysis of enzymatic reaction mechanisms is the QM/MM method. QM/MM compared to QM-cluster allows for the analysis of significantly larger systems, where a small portion is treated at the QM level, while force field-based methods are used for the rest of the system (Figure 5). This approach combines the advantages of QM calculations (accuracy, ability to describe chemical reactions) with those of MM methods (calculation speed). QM/MM was first proposed in 1976 by Warshel and Levitt [83], who, along with Karplus, were awarded the Nobel Prize in Chemistry for “the development of multiscale models for complex chemical systems”.

Similar to the QM-cluster method, QM/MM models can be based on both crystal structures and enzyme conformations obtained from MD simulations. The use of MD allows for sampling the conformational landscape of the enzyme, which is crucial since active site geometries may vary significantly depending on protein flexibility. Selecting representative conformations from MD trajectories ensures that the QM/MM calculations account for the dynamic nature of the enzyme and reduces the risk of bias resulting from relying on a single, possibly non-representative structure [84,85,86]. In addition, performing reliable QM/MM calculations involves an important choice regarding how to divide the system into QM and MM regions. The QM region includes the atoms of interest, such as amino acid residues in the active site, substrates, and cofactors, while the protein environment is treated at the MM level. The effect of QM region size on the accuracy of calculated enzymatic reaction parameters has been the subject of extensive recent research [87,88,89,90]. Although the QM/MM methodology seems to solve the problems of the QM-cluster approach, such as describing the missing part of the enzyme and choosing the ε constant, it faces its own challenges. There are three crucial factors to consider when studying enzymatic reaction mechanisms using the QM/MM approach: (i) how the interactions between the QM and MM regions are treated, (ii) the handling of covalent bonds crossing the QM/MM boundary, and (iii) the method used to compute the total energy of the system [91,92]. One of the most common methods for treating electrostatic interactions between QM and MM regions is electrostatic embedding [93]. This approach allows for polarization of the QM region, as the MM charge distribution is incorporated into the QM Hamiltonian during the calculations. To handle atoms at the boundary between the QM and MM regions, link atom approaches are commonly applied. In these methods, the free valences in the QM part are saturated with hydrogen atoms or methyl groups [94,95,96]. There are several approaches to calculating the total energy of a system in QM/MM methods, which can be grouped into additive and subtractive schemes. In the additive scheme, the interactions between both subsystems are treated explicitly (Equation (1)), while in the subtractive scheme, they are calculated at the MM level (Equation (2)). One of the most employed subtractive schemes is Own N-layer integrated molecular orbital molecular mechanics (ONIOM) [97,98,99].

(1) $$E_{total}=E_{QM}+E_{MM}+E_{QM-MM}$$

Equation (1): Total energy in the additive QM/MM scheme. $E_{total}$ is the total energy of the system, $E_{QM}$ is the quantum mechanical energy of the QM region, $E_{MM}$ is the molecular mechanics energy of the MM region, and $E_{QM-MM}$ represents the interactions between the QM and MM regions.

(2) $$E_{total}=E_{MM}^{full}+E_{QM}^{QM\;region}-E_{MM}^{QM\;region}$$

Equation (2): Total energy in the subtractive QM/MM scheme. $E_{total}$ is the total energy of the system, $E_{MM}^{full}$ is the MM energy of the entire system, $E_{QM}^{QM\;region}$ is the QM energy of the QM region, and $E_{MM}^{QM\;region}$ is the MM energy of the QM region subtracted to avoid double-counting.

Both methods, QM-cluster and QM/MM, are used in the analysis of enzymatic reaction mechanisms, including methylation reactions catalyzed by methyltransferases. There is an ongoing discussion about which of these approaches is better for this purpose. With a sufficiently large QM region, the most important interactions are explicitly included in the model, so both methods should result in comparable outcomes. Many studies have shown that energies and other reaction parameters converge faster with increasing QM region size for the QM/MM method compared to the QM-cluster method [100,101,102,103]. For a more comprehensive overview of the QM-cluster and QM/MM methodologies, readers are encouraged to consult recent review articles on the subject [104,105,106,107,108].

#### 1.3.2. What About Protein Dynamics? MD Simulations and QM/MM MD

The methods described so far do not take into account the fact that the structure of the enzyme complex is dynamic. One method that allows for the consideration of dynamic effects in the study of enzymatic reaction mechanisms is classical molecular dynamics simulations. The first MD simulations were conducted for gases in 1957 [109], while the first applications to proteins appeared in the late 1970s [110]. Research on MD simulations by Michael Levitt, Martin Karplus, and Arieh Warshel was also among the achievements that were honored with the Nobel Prize in Chemistry in 2013 [110,111,112].

The basis of MD simulation is the fact that by knowing the positions of the atoms in a molecule, it is possible to calculate the force acting on each atom. Then, using Newton’s equations, it is possible to predict the position of each atom over time. The resulting trajectory can be considered a 3-dimensional film that describes the dynamics of the analyzed biomolecule at the molecular level. Forces in MD simulations are calculated using a force field. Because typical systems simulated with MD contain around 100,000 atoms, it is not feasible to determine the system’s trajectory analytically. Instead, iterative numerical algorithms such as the Verlet algorithm [113] or the leapfrog method [114] are used to integrate the equations of motion. To avoid numerical instability, the time step should be lower than the fastest movements occurring in the system. Typical values used are 1 or 2 fs. For this reason, achieving simulation times on the order of microseconds requires performing 10^9^ steps. It is essential to recognize that the simulation time should be at least an order of magnitude longer than the characteristic timescale of the motion being studied. Therefore, commonly used durations on the order of 100 ns may not be adequate for all types of problems. Since the initial velocities of atoms are randomly selected according to the Maxwell–Boltzmann distribution, it is common practice to perform simulations in triplicate.

Force field-based methods, such as classical MD simulations, do not allow for the description of bond breaking between atoms. Consequently, their application to studying reaction mechanisms is limited. The vast majority of classical MD simulations are used to relax molecular structures, such as after docking to eliminate unfavorable contacts [17,88,115,116,117], as well as to study interactions with water molecules and ions [17,65,66,117], active site reorganization [65], and for studying protein–protein or ligand–protein interactions [117,118,119,120]. The biomolecule conformations obtained from these simulations are then used as a starting point for reaction mechanism calculations using QM-cluster and QM/MM approaches. The multitude of conformations is both an advantage and a disadvantage of this method. It is not possible to perform reaction mechanism calculations for every structure obtained. Consequently, it is necessary to limit the calculations to a small set of conformations, on the order of a few to a dozen. The most commonly used methods for selecting conformations for further mechanistic calculations include selection based on key distances [17,65,88,117], using the last structure from MD simulations [116], extracting snapshots at defined time intervals [121,122], and manual selection of snapshots [66].

Energy minimization methods, including QM-cluster and QM/MM, fail to take into account dynamic effects during a chemical reaction. Entropic and dynamic effects may play a significant role in assessing steric effects, analyzing different reaction pathways, or describing distinct protein conformations [123,124]. To account for these key effects, it becomes necessary to use a QM/MM MD approach. This approach combines the advantages of the QM/MM approach (description of chemical reactions) and MD simulations (dynamics-related effects). In the QM/MM MD approach, the system is divided similarly to the traditional QM/MM approach: the region important for the reaction, typically including the cofactor, substrate, catalytic residues, and water, is treated with quantum chemical methods, while the rest of the protein and water are described using force field methods. However, the QM/MM MD method does not focus on analyzing a single enzyme conformation but allows the system to evolve over time according to Newton’s laws of motion. Due to the high computational cost, QM/MM MD simulations are limited to short timescales, typically ranging from 10 to 1000 ps [125,126,127]. Because direct sampling of rare events such as bond breaking and chemical transformations can be inefficient on conventional timescales, enhanced sampling methods are frequently employed in QM/MM MD studies. These techniques facilitate the study of free energy surfaces (FES) associated with enzymatic reactions. The most popular sampling techniques are umbrella sampling and metadynamics.

Umbrella sampling is a biased sampling method in which a restraining potential is applied along a chosen reaction coordinate, such as a bond distance or angle, to enforce sampling of otherwise rare configurations. By performing multiple simulations with overlapping windows along the coordinate and subsequently reconstructing the probability distribution, it is possible to obtain the potential of mean force and accurate free energy profiles for the reaction. Metadynamics, in contrast, applies a time-dependent bias potential, usually in the form of Gaussian functions, that progressively fills free energy minima along selected collective variables. This procedure discourages the system from revisiting already explored states and drives it to overcome free energy barriers, thereby enabling efficient exploration of the energy landscape. The resulting bias potential can then be used to reconstruct the free energy surface of the studied enzymatic process.

Although the QM/MM MD method is significantly more complex and computationally demanding than QM-cluster and traditional QM/MM, it provides the most realistic picture of enzymatic catalysis, especially for systems where flexibility and entropy play an important role. Readers interested in exploring dynamic methodologies in greater detail are encouraged to consult [104,107,128,129,130].

#### 1.3.3. Common Pitfalls and Limitations of QM/MM Approaches

Despite their power, QM/MM methods have several inherent limitations that must be carefully addressed to obtain reliable results. One of the primary challenges in QM/MM studies is ensuring convergence of calculated properties with respect to the size of the QM region. Numerous studies have demonstrated that residues distant from the active site can still influence electrostatics and reaction energetics, meaning that too small a QM region may lead to underestimated barriers or incorrect mechanistic conclusions [87,88,89]. This convergence problem has therefore become a major focus of methodological work, with systematic QM region expansion often used to determine the minimal size yielding stable results. Boundary artifacts remain another challenge, as artificial link atoms or constraints can distort local geometry near the QM/MM interface. The choice and treatment of the solvent is another crucial factor affecting QM/MM accuracy. Simplified continuum models may underestimate electrostatic stabilization or fail to capture key water-mediated hydrogen bonds, whereas explicit solvent simulations provide a more realistic environment but at a higher computational cost [131]. Consequently, careful validation of solvent models and, where possible, inclusion of explicit water molecules in the QM region are recommended to ensure reliable free energy profiles and transition-state geometries. Another important limitation of QM/MM simulations is the timescale that can be feasibly accessed. Because the computational cost of QM/MM is high, typical trajectories are limited to tens or hundreds of picoseconds, whereas enzymatic reactions often occur on much slower timescales [125,126]. This discrepancy means that spontaneous bond-breaking events are rarely observed in unbiased simulations. To overcome this limitation, enhanced sampling techniques such as umbrella sampling or metadynamics are commonly applied. QM/MM methods are a powerful tool for studying complex chemical and enzymatic processes, but careful attention to QM region size, boundary effects, solvent treatment, and sampling timescales is essential.

#### 1.3.4. Mechanism-Guided Inhibitor Design in SAM-Dependent Methyltransferases

A detailed understanding of catalytic mechanisms provided by QM, QM/MM, and QM/MM MD approaches is not only valuable from a purely mechanistic perspective but also directly supports the rational design of selective inhibitors. Since SAM-dependent methyltransferases share conserved structural features, particularly within the SAM-binding pocket, achieving specificity is a major challenge [132]. Traditional inhibitors that mimic the cofactor or substrate often display limited selectivity, leading to off-target effects. By contrast, computational studies allow for the identification of subtle structural and electronic differences between closely related enzymes, which can be exploited to guide inhibitor design.

One particularly successful strategy was the development of transition state analogs. These inhibitors mimic the high-energy transition state in terms of both conformation and charge distribution. As a result, they can bind to their target enzyme with affinities that are several orders of magnitude higher than those of natural substrates, making them highly promising candidates for drug development across a wide range of therapeutic applications [133,134]. Computational methods make it possible to determine the structure of these short-lived transition states, thereby providing a rational basis for the design of inhibitors with substantially improved affinity and selectivity.

Recent examples highlight the successful synthesis of transition state analogues, which have demonstrated remarkable selectivity and potency. For example, transition state analogues of human phenylethanolamine N-methyltransferase were designed to replicate the geometry and electronic features of the S_N_2 methyl transfer transition state [135]. This approach yielded a tight-binding inhibitor with a K_i_ of 12.0 nM. Notably, it represents one of the first nanomolar transition-state analogue inhibitors reported for SAM-dependent methyltransferases. Recent studies have also reported the development of peptide-based transition state mimics targeting protein arginine N-methyltransferases (PRMTs) [136]. Using this strategy, histone H4 tail analogues were designed as transition state mimics, in which an adenosine moiety was covalently attached to the arginine residue, resulting in low micromolar IC_50_ values against PRMT1 and PRMT6. These results demonstrate the applicability of transition state mimicking strategies to the broader PRMT family, a group of enzymes increasingly recognized for their roles in cancer progression and therapeutic resistance [137].

Another emerging application of mechanism-guided design is the identification of previously unrecognized metal-ion binding pockets that can be exploited for inhibitor development. An illustrative example is research on the bacterial methyl transferase TrmD [66]. Computational analyses allowed the identification of a Mg^2+^ binding pocket in the active site, confirmed that TrmD’s catalytic mechanism depends on magnesium ions, and revealed how Mg^2+^ positions the SAM cofactor and active site residues to enable methyl transfer. This example demonstrates that computational identification of metal binding sites can guide the development of selective inhibitors, including potential antibacterial agents. Collectively, these studies underscore how mechanistic insights gained from computational studies, including transition state characterization and cation binding analysis, can be directly applied to the rational design of highly potent and selective inhibitors of SAM-dependent methyltransferases.

## 2. Computational Strategies in Methyltransferase Mechanism Studies

### 2.1. Investigating Catalytic Mechanisms Using Computational Models

The initial purpose for applying the QM-cluster approach in the study of methyltransferases was the investigation of reaction mechanisms. These investigations concentrated on both the ability of computational approaches to replicate the experimental reaction energy barrier and the elucidation of the reaction mechanism at the molecular level [138,139,140,141]. Nowadays, it is clear that these methods allow for obtaining reliable and repeatable results regarding catalyzed reactions.

One of the first studies including every aspect of the modern QM-cluster approach was the investigation of the histone lysine methyltransferase (HKMT) SET7/9 enzyme. This enzyme catalyzes the methylation of the N-terminal histone tail inside the chromatin structure [138]. In this study, the reaction mechanism of the HKMT-catalyzed reaction was investigated using four active site models of varying sizes (up to 132 atoms) derived from the 1O9S crystal structure [142]. The reaction energy barriers for the smallest Models A and B, measuring 18.8 and 21.7 kcal/mol, respectively, were comparable to the experimentally determined reaction energy barrier of 20.9 kcal/mol. However, the value of this barrier was sensitive to the selected dielectric constant and was lower by 1.5 and 2.6 kcal/mol, respectively, when using ε = 80. For the largest model, Model D, the reaction energy barrier was also comparable to the experimental barrier. Interestingly, compared to the smaller Models A and B, the reaction energy barrier was much less sensitive to the choice of the ε constant. This indicates that as additional groups are incorporated into the model, specifically as the truncation occurs further from the reacting moieties, the influence of the homogeneous solvation model decreases. The majority of the polarisation effects on reactive parts are already explicitly incorporated in the cluster models. Furthermore, even small models containing several dozen atoms allow the reaction barrier to be determined with reasonable accuracy.

**Figure 6:**
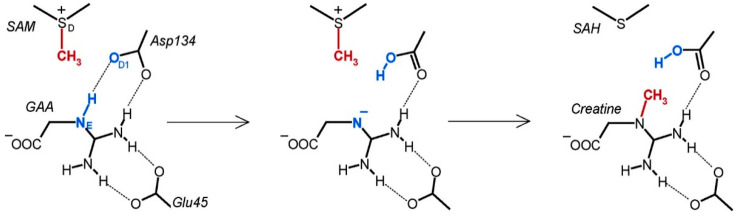
The originally proposed mechanism of guanidinoacetate methyltransferase. It involves deprotonation of the substrate by ASP134, followed by the transfer of the methyl group from SAM to guanidinoacetate, producing creatine and SAH. Reprinted with permission from [141]. Copyright 2025 American Chemical Society.

Another interesting case is guanidinoacetate methyltransferase (GAMT), which catalyses the final step of creatine biosynthesis by converting guanidinoacetate and SAM into creatine and SAH. The original proposed reaction mechanism involved the deprotonation of the substrate by ASP134, followed by the transfer of the methyl group, resulting in the product (Figure 6) [143]. The mechanism has been evaluated through a QM-cluster method utilising a single active site model comprising 92 atoms [141]. The first step of the proposed reaction mechanism was the proton transfer from guanidinoacetate to ASP134, examined through a linear transit scan that maintained a fixed distance between the proton and its acceptor, varying between 1.86 Å and 1.00 Å, while optimising all other degrees of freedom. Interestingly, the energy increase was monotonic, and no energy minimum was identified that corresponded to an intermediate where the proton is transferred to the aspartate. Afterwards, similar calculations were conducted regarding the transfer of the methyl group to the substrate, revealing an energy maximum at distances of 2.2–2.0 Å between the carbon atom of the methyl group and the nitrogen atom of the substrate, succeeded by a decrease in energy at shorter distances. At the same time, a proton transfer from the substrate to aspartate was observed. These findings suggest that the reaction mechanism catalysed by GAMT is concerted, involving an asynchronous transfer of the methyl group and proton. This contrasts with the previously proposed stepwise pathway, in which proton and methyl transfers were considered to occur in distinct, sequential steps. This study illustrates that the QM-cluster method is a powerful and versatile technique for examining complex reaction mechanisms, including those with multiple steps or concerted processes involving various functional groups.

The mechanism of the reaction catalyzed by GAMT was also analyzed based on the QM/MM approach [144]. In contrast to the previously discussed work, the authors decided to conduct the calculations using the conformation obtained from a 1 ns MD simulation, rather than directly based on the crystal structure. Interestingly, the QM/MM calculations also indicate that the reaction catalyzed by GAMT is occurring in a single step, involving both the transfer of the methyl group to the substrate and the transfer of the proton to ASP134. The reaction energy barriers for the concerted and stepwise mechanisms are 19.5 kcal/mol and 26.0 kcal/mol, respectively, and are in good agreement with the experimental barrier of 19.0 kcal/mol, as well as the barrier obtained in previous studies using the cluster approach [141]. Despite drawing a similar conclusion to Velichkova et al. [141] regarding the general course of the reaction catalyzed by this methyltransferase, noticeable differences can be observed in the proposed mechanisms. The transition state found for the simultaneous transfer of a methyl group and a proton, proposed based on the QM-cluster approach, was an asynchronous transition state. However, the use of the QM/MM approach with prior MD simulation leads to the identification of a synchronous transition state, in which both the bonds between the methyl group and the cofactor, as well as the proton and the substrate, are broken. The authors suggest that this difference may arise from the fact that in the case of QM/MM calculations, there were two water molecules in the active site of the enzyme that formed hydrogen bonds with the substrate, which were absent in the QM-cluster model [141].

This example shows that models based directly on the crystallographic structure may not accurately reflect certain details of the mechanism, and it may sometimes be necessary to physiologize the conformation of the enzyme complex, for example, by performing MD simulations before carrying out reaction mechanism calculations. Interestingly, this trend is noticeable in more contemporary works utilizing the QM-cluster approach, where the reaction mechanism calculations often rely on multiple enzyme conformations obtained from MD simulations [17,65,66]. In addition to computational approaches, experimental techniques such as 4D electron imaging have also proven effective in capturing protein dynamics, providing complementary insights into the structural changes and reaction dynamics [145].

### 2.2. Water Molecules as Catalytic and Structural Components

As previous research has shown, water molecules present in the active site of the enzyme may participate in the catalyzed reaction. One of the potential roles of water molecules is catalytic function. The catalytic mechanism is directly dependent on the presence of water molecules, as the reaction cannot proceed without them. One of the interesting examples is the reaction catalyzed by the Nucleolar Essential Protein 1 (Nep1).

**Figure 7:**
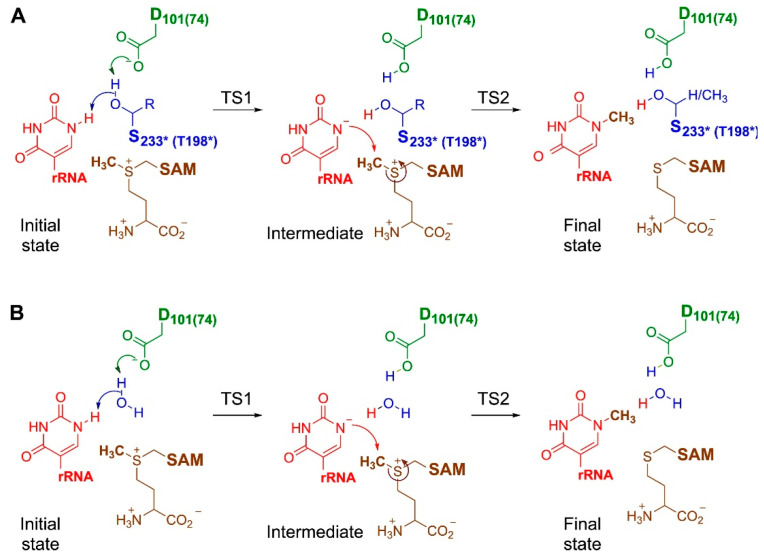
Panel (**A**): Originally proposed mechanism of pseudouridine methylation, where serine/threonine acts as a proton shuttle. Panel (**B**): Final mechanism involving a water molecule participating in deprotonation, resulting in a lower reaction energy barrier. Reprinted from [17], Copyright 2023, with permission from Elsevier.

Nep1 is an enzyme responsible for the N1 methylation of pseudouridine in archaeal 16S rRNA and eukaryotic 18S rRNA [146,147] and is crucial for ribosomal biogenesis and essential for the assembly of the small ribosomal subunit [148]. The original hypothesis regarding the mechanism of the catalyzed reaction assumed that the reaction proceeds in two chemical steps involving the deprotonation of pseudouridine by ASP101 and the transfer of a methyl group from SAM [17]. However, the details of the first step of the reaction were unclear due to the large distance between the proton acceptor and donor, which was 6.8 Å in the crystal structure of Nep1. Based on available crystallographic structures and the bioinformatics analysis of the Nep1 sequence from various organisms, the authors suggested that deprotonation involves an amino acid residue with a hydroxyl group (Figure 7A). However, this initially proposed mechanism turned out to be incorrect. MD simulations of Nep1 complexes from two distinct organisms revealed that water molecules may interact with the active site of the methyltransferase, bridging the N1 of pseudouridine and the proposed aspartate proton acceptor via two contiguous hydrogen bonds. Additionally, calculations using the QM-cluster approach showed that the methylation reaction involving a water molecule is associated with a lower reaction energy barrier than when the serine/threonine residue acts as a proton shuffle (Figure 7B). Furthermore, the reaction mechanism involving water molecules acting as catalysts was additionally confirmed by mutation analysis and reaction kinetics studies.

Water molecules involved in reactions catalyzed by methyltransferases may play not only a catalytic role, but also a structural one. Structural water in enzymes may be responsible for the stability or flexibility of the active site, the orientation of ligands or amino acid residues, as well as the maintenance and stabilization of the fold [149,150,151]. One of the methyltransferases containing structural water in the active site is loganic acid methyltransferase (LAMT).

LAMT is an enzyme that catalyzes the transfer of a methyl group from the SAM cofactor to loganic acid, resulting in the formation of loganin. This methyltransferase thus participates in the biosynthetic pathway of vincristine and vinblastine, valuable anticancer drugs [152,153]. One of the approaches to investigating the reaction mechanism catalyzed by LAMT involved analysis of its crystal structure, site-directed mutagenesis, and reaction kinetics [154]. Mutational analysis indicated that amino acid residues such as GLN38, HIS162, and TRP163 are crucial for the catalytic activity of LAMT and may be responsible for substrate recognition and positioning. Both HIS162 and TRP163 were shown in the crystal structure to form hydrogen bonds with loganic acid, which further reinforced the conclusions drawn from the analysis of mutated variants of the methyltransferase. In contrast, the GLN38 residue was beyond the range of hydrogen bonding interactions with the substrate. Nevertheless, based on mutational analysis and the conservation of GLN38 in the sequences of other carboxylic acids O-methyltransferases, the authors concluded that this residue may still be responsible for substrate binding. This observation prompted further investigation into the mechanism of the reaction catalyzed by LAMT, which served as a key motivation for the application of computational methods.

**Figure 8:**
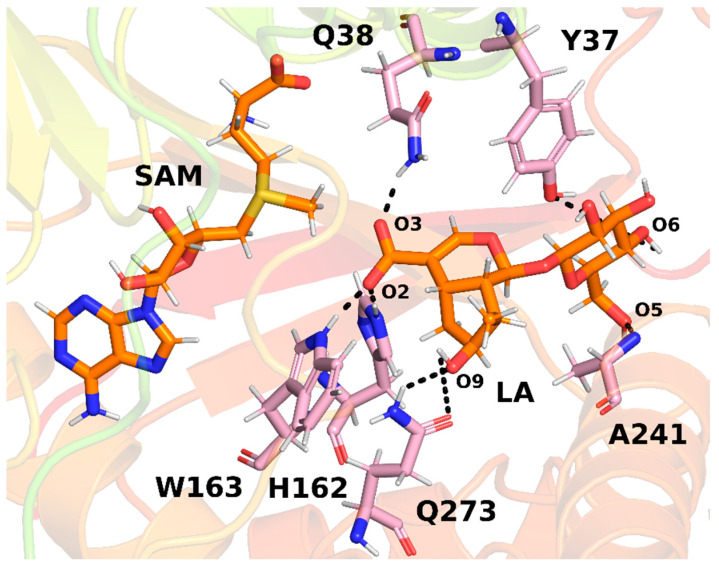
The most frequently observed hydrogen bonds between loganic acid (LA) and the protein in MD simulations, shown on an example structure. Dashed lines indicate bonds between specific atoms. Adapted from [65], licensed under CC BY 4.0.

Interactions between LAMT and loganic acid were analyzed using MD simulations [65]. During the simulations, all three residues, HIS162, TRP163, and GLN38, formed stable hydrogen bonds (Figure 8). This observation not only confirmed the role of HIS162 and TRP163 in substrate binding but also revealed the previously unclear role of GLN38 in the course of the catalyzed reaction. Interestingly, it has been identified that TRP163 forms not only direct hydrogen bonds with loganic acid, observed in the crystal structure of LAMT methyltransferase, but also indirect ones, involving a water bridge. For this reason, it was concluded that TRP163 may not only be responsible for substrate binding and orientation but also for recognizing the substituted iridoid ring, as the bridging water interacted simultaneously with the substrate’s carboxyl group and the ring’s hydroxyl group. Further analyses of the reaction mechanism using the QM-cluster approach confirmed that both conformations of the active site, with the presence of a water bridge and with direct hydrogen bonding between the substrate and the TRP163 residue, favor the methylation reaction and exhibit a similar reaction energy barrier. As the conclusion of in silico studies, two interaction patterns between loganic acid and the LAMT methyltransferase were identified, and the structural role of a water molecule in the active site was postulated.

The examples discussed here show that computational methods allow for the analysis of the role of water molecules in the mechanism of reactions catalyzed by methyltransferases. Importantly, in these studies, it was necessary to use MD simulations to obtain enzyme conformations similar to those observed under physiological conditions. For this reason, one should be cautious when conducting reaction mechanism calculations based directly on the crystallographic structure without prior MD simulation, as this may result in erroneous conclusions or an inadequate representation of the reaction mechanism.

### 2.3. Functional Role of Metal Ions in Methylation Reactions

Based on previous discussions regarding the role of MD simulations in reproducing the physiological conformations of methyltransferases, it is also important to consider the role of metal cations in the catalytic activity of this group of enzymes. While the catalytic activity of most methyltransferases is independent of the presence of specific metal cations, some of them bind ions such as Mg^2+^ to catalyze the transfer of the methyl group [66,155,156,157]. The identification of ion binding sites is important not only for understanding the methylation mechanism but also from the perspective of drug design, as small ligands can competitively bind in these pockets.

**Figure 9:**
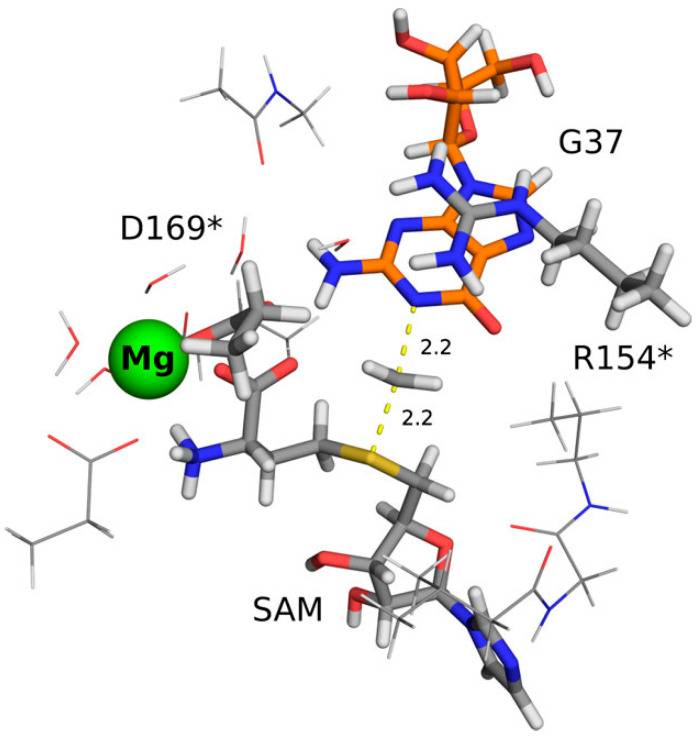
Optimized transition state structure for the methyl transfer catalyzed by the Mg^2+^-dependent TrmD methyltransferase. Mg^2+^ is located in the negatively charged binding pocket, stabilizing the bent conformation of the cofactor. Adapted from [66], licensed under CC BY 4.0.

One of the interesting examples of a Mg^2+^-dependent methyltransferase is the TrmD methyltransferase. TrmD is a knotted SPOUT methyltransferase [158,159] responsible for the production of m^1^G37-tRNA, and consequently, it is necessary for maintaining the correct reading frame during protein biosynthesis and thus essential for life [160,161,162,163]. Surprisingly, experimental studies showed that the transfer of the methyl group by bacterial TrmD requires the presence of Mg^2+^ cations [164]. However, what was not obvious was the exact binding site of these ions and their role in the reaction mechanism. Considering that computational methods are well-suited for studying ions such as Mg^2+^, which are spectroscopically silent and often difficult to distinguish from water molecules in electron density maps [165,166], Perlinska et al. conducted MD simulations and QM calculations to better understand the mechanism of the reaction catalyzed by TrmD [66]. MD simulations showed that Mg^2+^ preferentially binds to the negatively charged binding pocket in the active site of TrmD. The binding of the cation induces conformational changes not only in the catalytic amino acid residues but also stabilizes the bent conformation of the cofactor S-adenosylmethionine [167]. Interestingly, QM calculations showed that only the enzyme conformations with bound Mg^2+^ are catalytically competent, due to the plausible energy barrier for the methyl transfer reaction (Figure 9). The obtained results were further validated through experimental mutation studies, which additionally confirmed the role of amino acid residues binding Mg^2+^ in enzymatic catalysis. This work has shown that the combination of MD simulations and QM calculations is an effective tool for studying the role of ions and identifying their binding sites. Moreover, the newly identified negatively charged pocket binding Mg^2+^ could become a new target in the design of TrmD inhibitors.

Similar to the TrmD methyltransferase, the catalytic activity of the catechol-O-methyltransferase (COMT) is also dependent on the presence of Mg^2+^ cations [155,157]. COMT is an enzyme that degrades catecholamines such as dopamine, epinephrine, and norepinephrine, as well as a variety of drugs and substances containing a catechol structure. Some COMT substrates are neurotransmitters, and for this reason, dysfunctions of this methyltransferase are associated with diseases such as Parkinson’s disease, schizophrenia, anxiety disorders, or substance abuse. COMT catalyzes the transfer of a methyl group from the cofactor SAM to one of the hydroxyl groups of catechol derivatives in the presence of divalent cations. COMT under physiological conditions binds the Mg^2+^ cation, whose role is to maintain the substrate in a reactive orientation relative to the cofactor [168,169,170]. Considering the seemingly insignificant role of Mg^2+^ in the reaction mechanism, it remained unclear why substitution of this cation with other cations sometimes leads to the inhibition of this enzyme’s activity (Ca^2+^, Sn^2+^, Fe^3+^, Ni^2+^), while in other cases it slightly reduces the enzyme’s activity (Cd^2+^, Fe^2+^, Zn^2+^) or even causes an increase in activity (Mn^2+^, Co^2+^) [157].

This subject area was examined by Sparta et al., who used the QM/DMD approach to investigate the impact of various cations, such as Mg^2+^, Ca^2+^, Fe^2+^, and Fe^3+^ on the activity of COMT methyltransferase [171]. QM/DMD simulations showed that the architecture of the enzyme’s active site was strictly dependent on the bound ion. For the cation Mg^2+^, which is bound under physiological conditions, it was similar to that observed in the available crystallographic structures. It shows that the QM/DMD method allows for the reconstruction of the native structure of COMT complexes. Interestingly, the substitution of the Mg^2+^ cation with Fe^2+^ does not lead to significant changes in the conformation of the enzyme’s active site. Similarly, the substitution with Fe^3+^ maintains the key features of the native active site and only leads to a decrease in some bond lengths between the ligand and the metal cation. Interestingly, earlier experimental studies have shown that the Fe^2+^ cation causes a slight decrease in the activity of the COMT methyltransferase, where Fe^3+^ is its inhibitor [157,172]. This is reflected in the reaction barriers for the transfer of the methyl group from mechanistic studies. In the case of the COMT complex with Fe^2+^, the reaction barrier is similar to that for the Mg^2+^ cation, while for Fe^3+^, it is significantly higher. This shows that the mechanism of Fe^3+^ inhibition is more related to an electronic effect rather than the rearrangement of the enzyme’s active site. In contrast to the previously discussed ions, the binding of the Ca^2+^ cation has a significant impact on the conformation of COMT. QM/DMD simulations revealed that in the presence of Ca^2+^, the reagents adopt a less favorable configuration for the methyl group transfer reaction. The authors emphasize that this effect, along with the increase in reaction energy observed in the reaction mechanism calculations, is responsible for the inhibitory effect of this cation on COMT activity.

The examples discussed here highlight the significant role of in silico methods in studying Mg^2+^-dependent methyltransferases. Computational approaches not only help identify previously unknown cation binding sites provide insight into their influence on the reaction mechanism. They also enable an understanding of how substituting native cations can affect methyltransferase activity. Such studies enhance our understanding of experimental data, as demonstrated by the case of COMT, and, perhaps more importantly, provide essential guidelines for the design of future experiments, including those intended to validate newly discovered cation binding sites.

### 2.4. Computational Insights into Substrate Specificity

Methyltransferases can exhibit not only selective binding to specific cations but also distinct substrate specificity. Some of these enzymes methylate specific substrates, while others have a broader substrate spectrum and only require the presence of a particular group [173,174]. Among the enzymes with narrow substrate specificity are TrmD and Nep1, which methylate specific nitrogenous bases such as guanine in tRNA and pseudouridine in rRNA, respectively [147,175]. In contrast, enzymes such as COMT and thiopurine methyltransferase act on a broader range of substrates [157,176,177]. Understanding the basis of substrate specificity is important for understanding the catalytic activity of methyltransferases, but it may also allow for the rational design of enzymes with altered selectivity. One example demonstrating the application of computational methods in studying the substrate specificity of methyltransferases is the research on BezA methyltransferase [118].

**Figure 10:**
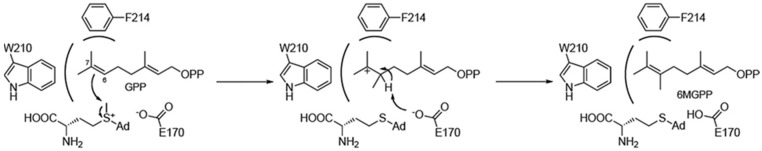
Proposed reaction mechanism of BezA based on ONIOM calculations and the MD-derived structure. The methyl group is first transferred to the C6 atom of GPP, forming a carbocationic intermediate, which is subsequently deprotonated with the involvement of GLU170. Used with permission of Mateusz Jędrzejewski, from [118]; permission conveyed through Copyright Clearance Center, Inc.

Methyltransferase BezA is an enzyme that catalyzes the transfer of a methyl group to the C6 atom of geranyl pyrophosphate (GPP) in the presence of Mg^2+^ cations [118]. This enzyme is responsible for expanding the structural diversity of terpenoids through the synthesis of methylgeranyl pyrophosphate (MGPP) in the benzastatin biosynthetic pathway [178]. Derivatives of benzastatin are natural compounds isolated from *Streptomyces* species [179,180,181,182,183,184,185,186], with interesting biological properties such as neuroprotective and antiviral activities [180,183]. Considering the biological activity of these biosynthesis products, it is intriguing to engineer mutated variants of BezA that would generate “unnatural” natural products. To accomplish this goal, Tsusumi et al. investigated the mechanism of catalytic activity of BezA methyltransferase using QM/MM calculations, MD simulations, and rational mutagenesis [118]. ONIOM calculations performed on the structure obtained from MD simulations showed that the reaction catalyzed by BezA is a two-step reaction. In the first step, the transfer of the methyl group to the C6 atom of GPP occurs, resulting in the formation of a carbocationic intermediate. This intermediate is then deprotonated with the involvement of the GLU170 residue (Figure 10). The proposed mechanism is consistent with the experimental results obtained by the authors, which indicated that the BezA GLU170GLN variant does not catalyze the methylation reaction. Next, the authors, based on a comparative analysis of the structures of BezA and GPP C2-methyltransferase, proposed that TRP210 is responsible for narrowing the ligand-binding pocket and thus for the substrate specificity of BezA methyltransferase. This led to the hypothesis that replacing TRP210 with a smaller amino acid could change the enzyme’s specificity, allowing it to accept a larger substrate, such as farnesyl pyrophosphate (FPP). Interestingly, the BezA TRP210ALA variant did not methylate its natural substrate, GPP, but instead exhibited altered substrate selectivity by methylating FPP. To gain additional insight into the reaction mechanism of the mutated enzyme variant, MD simulations and QM/MM calculations were conducted. The obtained results showed that the rest of the FPP spontaneously folded into the region occupied by the indole moiety of TRP210 and formed a stable complex with the BezA TRP210ALA variant. The energy barrier for the FPP methylation reaction was 0.7 kcal/mol higher than for the GPP methylation reaction by wild-type BezA, which is consistent with reaction kinetics studies indicating that the TRP210 mutation reduces catalytic efficiency.

As the previous example showed, computational methods allow for a better understanding of the substrate specificity of methyltransferases and serve as a valuable tool in the rational design of enzymes. One of the most important families of methyltransferases, which are significant from the perspective of enzyme design and their application in the biotechnology industry, is the SABATH family [187]. Enzymes from this family are involved in the biosynthesis of plant secondary metabolites, responsible for attracting pollinators, defensive functions, and disease resistance [188]. Some of the products of biosynthetic pathways involving SABATH methyltransferases, such as vincristine and vinblastine, are valuable therapeutic agents [152,189]. Additionally, it has been shown that a small modification in the SABATH protein sequence can lead to a change in substrate specificity [4,190,191,192]. Consequently, these enzymes hold significant potential as biocatalysts in the pharmaceutical industry [193].

One of the best-studied methyltransferases from the SABATH family is salicylic acid methyltransferase (SAMT). SAMT catalyzes the transfer of a methyl group from SAM to one of the oxygen atoms of the carboxyl group of salicylic acid, resulting in the formation of methyl salicylate. Earlier experimental studies reveal that the catalytic efficiency of SAMT is significantly lower for 4-hydroxybenzoic acid (4HA) compared to salicylic acid (SA) [194,195,196,197]. The reason for the significant reduction in activity resulting from the alteration of the hydroxyl group from the 2-position (as in SA) to the 4-position (as in 4HA) remained unclear. To understand the substrate specificity of the SAMT methyltransferase, Yao et al. conducted QM/MM MD simulations [198]. Computational results indicated that the energy barrier for methyl group transfer to 4HA is approximately 5 kcal/mol higher than that for the natural substrate, which is consistent with previous experimental studies [194,195,196,197]. In the simulations for the SAMT complex with SA, the reagents before the reaction adopted a configuration similar to that observed in the transition state for the transfer of the methyl group. The exchange of the natural substrate with 4HA resulted in a significant distortion of the orientation of the reagents compared to the enzyme complex with SA. For this reason, generating a structure similar to the transitional state requires a significant change in the configuration of SAM and 4HA. This conformational change requires additional energy, which contributes to an increased reaction barrier and reduced catalytic efficiency of the enzyme in the case of 4HA methylation. These insights demonstrate the essential effect of substrate orientation and active site configuration on the catalytic activity and specificity of SAMT.

**Figure 11:**
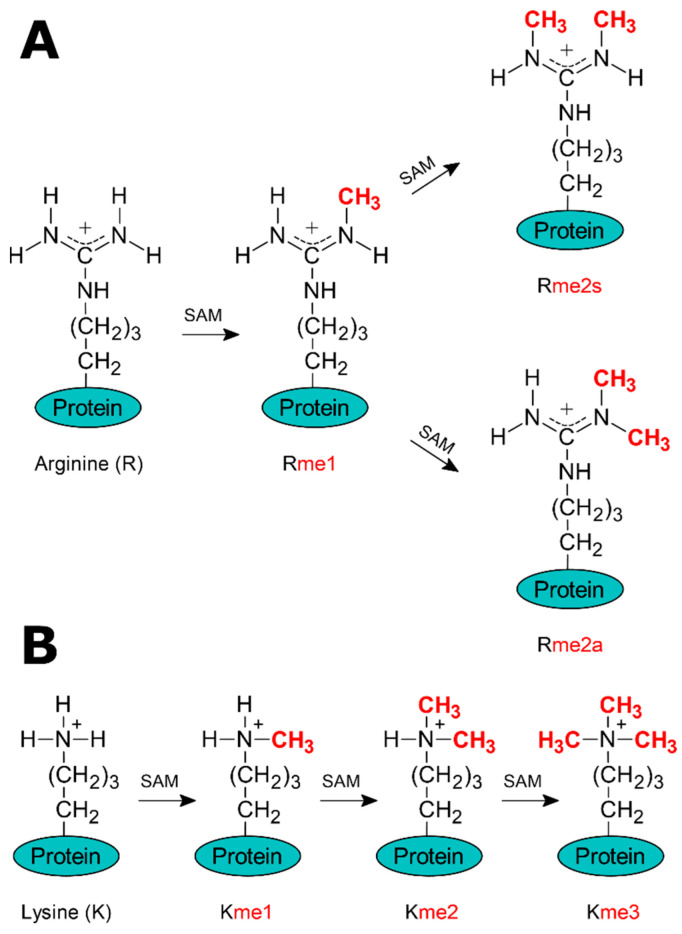
Methylation of arginine (**A**) and lysine (**B**) residues catalyzed by SAM-dependent methyltransferases: (**A**) Arginine undergoes sequential methylation to form monomethylarginine (Rme1), symmetric dimethylarginine (Rme2s), or asymmetric dimethylarginine (Rme2a). (**B**) Lysine is progressively methylated to mono- (Kme1), di- (Kme2), and trimethyllysine (Kme3). The transferred methyl groups are highlighted in red.

In contrast to previous examples focused on substrate specificity towards different molecules and monomethylation reactions, it is equally important to understand how methyltransferases distinguish the degree of methylation, leading to mono-, di-, or trimethylation of the same residual substrate. This type of specificity is important in the context of histone modifications, such as lysine and arginine methylation [199]. Histone methylation plays an important role in various biological processes, including gene expression, DNA damage response, cell cycle regulation, stress response, development, and differentiation [200,201,202]. Lysines can undergo monomethylation, dimethylation [203], or trimethylation [204] on their ε-amino group (Figure 11B), while arginines can be monomethylated [205], symmetrically dimethylated, or asymmetrically dimethylated on their guanidino group (Figure 11A) [206]. The issue of lysine and arginine methylation in histones has been extensively studied using computational methods [121,122,127,138,207,208,209,210,211,212,213,214,215,216,217]. One of the interesting examples of the application of QM/MM MD simulations was the research on the catalytic activity of the PRMT7 methyltransferase [209].

PRMT7 was originally identified as an enzyme catalyzing the monomethylation of arginine [218]. The substrate specificity of this methyltransferase has been a subject of controversy, as Lee et al. have shown that it also produces symmetric dimethylarginine [219]. One of the potential explanations for these discrepancies was the contamination of PRMT7 with the methyltransferase PRMT5 during purification, which would be responsible for the formation of dimethylarginine [220,221]. Subsequent experiments showed that PRMT7 is solely responsible for monomethylation, contrary to previous in vitro studies [222,223,224,225]. The catalytic mechanism and substrate specificity of PRMT7 were also studied using computational methods [209]. First, the authors focused on analyzing the monomethylation reaction with the substrate arginine. In silico studies have shown that the catalytic mechanism of PRMT7 methyltransferase is associated with the formation of reactive (near attack) substrate conformations and the increase in arginine nucleophilicity through the modification of charge distribution via substrate interactions with the active site. The authors additionally highlight the role of residues GLU172 and GLU181 in stabilizing the transition state for the methyl group transfer. It is interesting that the side chains of these amino acid residues also serve as proton acceptors in the second step of the reaction, and the transfer of protons to each of these residues occurs with practically equal probability. The second methylation by PRMT7 was also investigated. The free energy barriers for the transfer of the second methyl group were found to be generally high, aligning with experimental studies indicating that PRMT7 functions as a monomethyltransferase. Interestingly, studies on PRMT7 mutants showed that the GLU181ASP/GLN329ALA variant can produce symmetric dimethylarginine similarly to the previously mentioned methyltransferase PRMT5 [127,226]. Similar calculations using QM/MM MD simulations were conducted by another group for the previously mentioned methyltransferase PRMT5 [227]. Yue et al. investigated both the monomethylation and dimethylation reactions of arginine involving the PRMT5 methyltransferase. Calculations have shown that PRMT5 can catalyze the formation of not only methylarginine but also symmetric dimethylarginine, which is associated with an energy barrier of 19–20 kcal/mol and 18–19 kcal/mol, respectively. Interestingly, both in the case of the first and second methylation, the GLU435 residue acts as a proton acceptor. These calculations are consistent with experimental evidence indicating that PRMT5 functions as a symmetric arginine dimethyltransferase [228]. Moreover, the estimated theoretical barriers are consistent with the experimental energy barrier of the reaction, which is approximately 18 kcal/mol [229].

These computational studies demonstrate the effectiveness of in silico methods in elucidating the catalytic mechanism and verifying the substrate specificity of methyltransferases, particularly in resolving discrepancies between experimental observations. Their findings show how different amino acid residues in the active site can influence the substrate specificity of methyltransferases. Such approaches can not only complement experimental results but also allow for the prediction of enzyme functions and serve as a guide in planning future experiments.

## 3. Conclusions

The wide range of examples presented in this review clearly demonstrates the growing role of computational methods in studying SAM-dependent methyltransferases. The application of the QM-cluster approach, hybrid QM/MM, and MD simulations enables a detailed analysis of enzymatic reaction mechanisms at the atomic level, including short-lived transition states that are often inaccessible to experimental techniques. These approaches not only provide insights into the methyl group transfer process but also allow for the evaluation of the roles of water molecules, metal ions, and active-site residues in catalysis. Furthermore, computational models have been successfully used to explain substrate specificity, catalytic selectivity, and the effects of enzyme mutations. The reviewed studies also highlight how structural flexibility and protein dynamics, addressed through MD simulations and QM/MM MD methods, affect enzymatic activity and reaction pathways. Collectively, the evidence presented underscores that in silico methods are now essential tools in enzymology, complementing and extending experimental findings. They support the rational design of selective inhibitors and engineered enzymes with tailored properties, paving the way for their application in drug discovery, synthetic biology, and biotechnology. Mechanistic insights into methyltransferases can also guide drug discovery by supporting the design of transition-state analog inhibitors and small molecules targeting metal-ion binding sites. These strategies improve selectivity, minimize off-target effects, and open new opportunities for therapeutic intervention in diseases linked to abnormal methylation.

## Data Availability

Not applicable.

## Supplementary Materials

The following supporting information can be downloaded at: https://www.mdpi.com/article/10.3390/ijms26189204/s1. References [17,65,66,70,72,87,88,115,116,117,118,119,120,121,122,125,126,127,138,139,140,141,144,171,198,207,208,209,210,211,212,213,214,215,216,217,227,230,231,232,233,234,235,236,237,238,239,240,241,242,243,244,245,246,247,248,249] were cited in Supplementary Materials.
